# Characterization of microbial communities in anaerobic acidification reactors fed with casein and/or lactose

**DOI:** 10.1007/s00253-022-12132-5

**Published:** 2022-08-26

**Authors:** Zhe Deng, Ana Lucia Morgado Ferreira, Henri Spanjers, Jules B. van Lier

**Affiliations:** 1 Department of Water Management, Delft University of Technology, Stevinweg 1, 2628 CN Delft, the Netherlands; 2 Veolia Water Technologies Techno Center Netherlands B.V. - Biothane, Tanthofdreef 21, 2623 EW Delft, The Netherlands

**Keywords:** Anaerobic digestion, Carbohydrates–proteins, Microbial community, Protein degraders, Protein-rich waste

## Abstract

**Abstract:**

Protein-rich agro-industrial waste streams are high in organic load and represent a major environmental problem. Anaerobic digestion is an established technology to treat these streams; however, retardation of protein degradation is frequently observed when carbohydrates are present. This study investigated the mechanism of the retardation by manipulating the carbon source fed to a complex anaerobic microbiota and linking the reactor performance to the variation of the microbial community. Two anaerobic acidification reactors were first acclimated either to casein (CAS reactor) or lactose (LAC reactor), and then fed with mixtures of casein and lactose. Results showed that when lactose was present, the microbial community acclimated to casein shifted from mainly *Chloroflexi* to *Proteobacteria* and *Firmicutes*, the degree of deamination in the CAS reactor decreased from 77 to 15%, and the VFA production decreased from 75 to 34% of the effluent COD. A decrease of 75% in protease activity and 90% in deamination activity of the microbiota was also observed. The microorganisms that can ferment both proteins and carbohydrates were predominant in the microbial community, and from a thermodynamical point of view, they consumed carbohydrates prior to proteins. The frequently observed negative effect of carbohydrates on protein degradation can be mainly attributed to the substrate preference of these populations.

**Keypoints:**

• *The presence of lactose shifted the microbial community and retarded anaerobic protein degradation.*

• *Facultative genera were dominant in the presence and absence of lactose.*

• *Substrate-preference caused retardation of anaerobic protein degradation.*

**Supplementary Information:**

The online version contains supplementary material available at 10.1007/s00253-022-12132-5.

## Introduction

Proteins are complex molecules that can contain more than 50 amino acids. They can be roughly divided into insoluble fibroid proteins and soluble globular proteins. The fibroid proteins, e.g. in manure fibres, are more resistant to hydrolysis due to the lower solubility than globular proteins (Sanders 2001). Proteins are abundantly found in food processing industries, for example, dairy and meat, and therefore end up in waste and wastewaters of dairy, slaughterhouse, and fishery industries, as well as in food residuals (Braguglia et al. 2018; Wang et al. 2005). Being an important component of cells, proteins are also found in biomass, such as algae and spent biomass of biological treatment processes (Ganesh Saratale et al. 2018; Magdalena et al. 2018; Mata-Alvarez et al. 2014).

These protein-rich waste streams are usually high in organic load and represent a major environmental problem, especially regarding the growth of the global population and increasing demand for food protein (Bustillo-Lecompte and Mehrvar 2015; Hassan and Nelson 2012). Direct discharge of protein-rich waste streams into surface water can lead to severe damage to the water quality, i.e., causing eutrophication and aquatic life extinction (Baker et al. 2021). The stricter discharge standards and the decreasing availability of freshwater contribute to the requirement for both wastewater treatment and resources recycling (Bustillo-Lecompte and Mehrvar 2015). Due to the high organic load, the protein-rich waste streams are preferably treated by anaerobic digestion (AD), which can purify the wastewater by converting the organic matters to a gaseous energy carrier, i.e., biogas (Adhikari et al. 2018).

Anaerobic protein degradation generally consists of four steps, which are hydrolysis of protein to amino acids, deamination and acidogenesis of amino acids to ammonium and VFAs, acetogenesis of VFAs to acetic acid, and methanogenesis (Hassan and Nelson 2012). Previous studies found that protein hydrolysis is slower than that of carbohydrates, and its efficiency decreases with the increase in the availability of carbohydrates (Wang et al. 2014; Yu and Fang 2001). The mechanism of this impact is unclear. Although the Stickland reaction pathways and involved microorganisms were reported in the 1960s (Barker 1961), the conclusions were based on studies on culturable bacteria that account for a small fraction of the total diversity (Stewart 2012). Generally, it is believed that in an AD reactor, there is a specific group that converts proteins (Barker 1961), and this group is reported to have a slower growth rate than the carbohydrates fermenting bacteria, i.e., protein-fermenting microbiota μ_max_ = 0.08—0.15 d^−1^, carbohydrates-fermenting microbiota μ_max_ = 0.3—1.25 h^−1^ (Pavlostathis and Giraldo-Gomez 1991; Tang et al. 2005). There is a lack of information to identify and characterize the protein-fermenting microorganisms (hereafter referred to as protein degraders) and to link the microbial process performance with the microbial community during anaerobic protein conversion.

With the development of genetic technologies, it is possible to analyse the entire microbial community and identify the dominant microorganisms. In our present study, we aimed to identify the dominant microorganisms under three circumstances, i.e., protein as the sole substrate, carbohydrate as the sole substrate, and a mixture of protein and carbohydrate as the substrate. In the experiments, we used casein and lactose as substrates representing proteins and carbohydrates, respectively. By characterizing the dominating genera and their ability to ferment proteins/carbohydrates, we intended to reveal the mechanism of the carbohydrates’ impact on the anaerobic protein degradation in a complex microbiota. These results will lead to a better understanding of anaerobic protein degradation at a microbial level, and would support the future design and operations of anaerobic digestion reactors for the treatment of protein-rich waste streams.

## Materials and methods

### Materials

Complex microbiota from an anaerobic membrane bioreactor (AnMBR) fed with protein- and lipids-rich dairy wastewater was used as inoculum, the inoculum was taken at Biothane—Veolia Water Technologies Techno Center Netherlands B.V Research Facilities, in Delft, The Netherlands. The volatile suspended solids (VSS) content of the inoculum was 14.5 g VSS·kg^−1^ wet weight, and 3 g VSS was added to each reactor (working volume of 2 L) to achieve an initial microbial concentration of 1.5 g VSS·L^−1^.

Three substrates were used in the experiment: A) casein (Fisher Scientific), with casein as the sole carbon source, B) lactose (Sigma Aldrich), with lactose as the sole carbon source, and C) mixture of casein and lactose as carbon sources, further referred to as MIX, with a ratio of 50%: 50% in terms of chemical oxygen demand (COD). The addition of nutrients was based on the anaerobic fermentation process growth media protocol of Hendriks et al. (2018). The substrates contained 6000 ± 100 mg COD·L^−1^ and 800 ± 50 mg N·L^−1^.

### Operation of acidification reactors

**Fig. 1:**
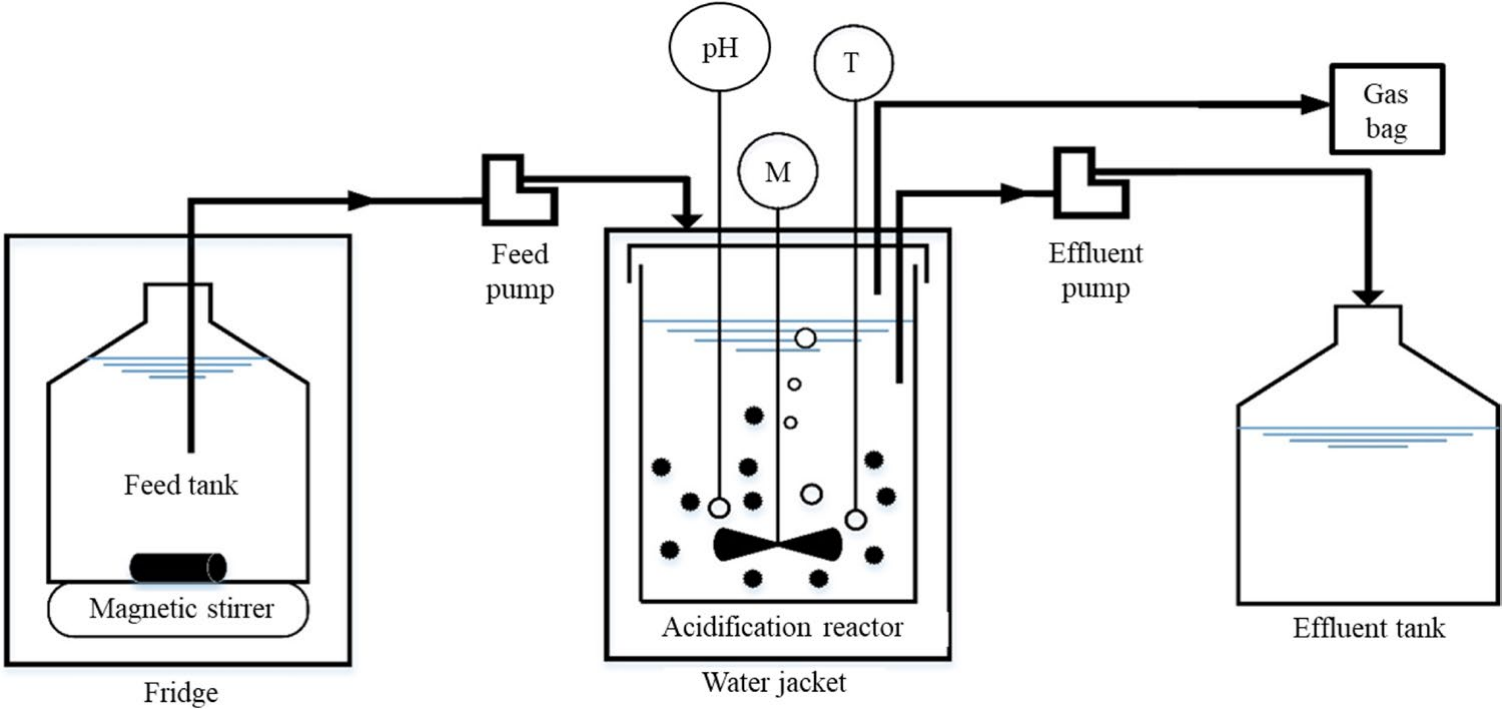
Schematic diagram of the reactor set-up

The reactors were mounted in parallel as shown in Fig. 1, each reactor set-up had a feed tank, a 3.5 L double layer (water jacketed) glass vessel, and an effluent tank. The feed tanks were stored in a fridge at 4 °C to minimize biological processes. The two vessels of 2 L working volume were operated as acidification reactors at 25 ± 3 °C and an HRT of 24 h. The temperature in the acidification reactors was controlled by using a recirculating water bath (Thermo Haake DC10), the pH in each tank was measured with a probe (Hamilton pH sensor), and a controller (Hach SC200 Universal Controller, USA) was used to control the pH in both reactors at 6 ± 0.2 by adding 0.5 mol·L^−1^ NaOH (Yu and Fang 2003). The volumetric loading rate (VLR) was 8 kg COD·m^−3^·d^−1^.

The operation of the reactors was divided into two stages, stage I lasted 78 days (days 7–84) and stage II lasted 48 days (days 85–132). In the first stage, the CAS reactor, fed with substrate A) casein, was acclimated to pure protein substrate, whereas the LAC reactor, fed with substrate B) lactose, was acclimated to pure carbohydrate substrate. At the beginning of stage II, the feed of both reactors (CAS and LAC) was switched to substrate C) the mixture of casein and lactose (MIX), and the microbiota was acclimated to the mixture of protein and carbohydrate. Magnetic stirrers were used to ensure the mixing of the feed, while two stirring blades connected to motors were used for mixing the reactors at 50 rpm. Four peristaltic pumps (Longer L100-18–2 with FG25-13 pump head, China) were used for influent feeding and effluent extraction. The operational conditions were constant during all stages, controlled by LabVIEW.

Effluent samples were taken twice a week, and the reactors were considered stable when the difference between daily feed COD and effluent COD was less than 2% and the variation in total VFA production and deamination was less than 10% in a week. The reactors were operated for 132 days. To acclimate the microbiota towards a specific substrate, stage I lasted 84 days, whereas stage II started from day 86 and lasted 46 days.

### Deamination activity tests

To investigate the deamination activity of the microbiota, batch tests were carried out in duplicates with microbiota samples taken from the reactor at the end of each stage. Glass bottles with a total volume of 350 mL were used for the batch test. The microbiota concentration in each bottle was 1.5 g VSS·L^−1^. Substrate A) of 190 ± 0.2 mL and buffer solution (3.5 g NaHCO_3_·L^−1^) was added to achieve a total liquid volume of 200 ± 0.5 mL. All bottles were incubated at 25 °C, with manual shaking before sampling. Liquid samples of 2 mL were taken from each bottle with syringes at 0 h, 1 h, 4 h, 8 h, 12 h, 20 h, and 24 h, and COD, total Kjeldahl nitrogen (TKN), VFAs, and ammonium (NH_4_^+^) were measured.

### Analytical methods

The solids contents (TSS and VSS), TKN, and NH_4_^+^ of the reactor effluent were analysed following standard methods (APHA 1998). The COD measurements were done with HACH-Lange kits (LCK014) without pre-treatment. For VFA measurement, samples were centrifuged at 13,500 × g for 5 min and filtered through 0.45 μm membrane filters (Whatman, Sigma Aldrich). VFAs, including acetic acid (C2), propionic acid (C3), iso-butyric acid (iC4), butyric acid (C4), iso-valeric acid (iC5), valeric acid (C5), and hexanoic acid (C6), were measured by gas chromatography (GC, Agilent Technologies 7820A) equipped with a CP 7614 column (WCOT Fused Silica 25 m × 0.55 mm, CP-wax 58 FFAP capillary, Agilent Technologies) and flame ionization detector. The injector temperature was 250 °C, and nitrogen gas (28.5 mL·min^−1^) with a split ratio of 10 was used as a carrier. The GC oven method sequence was started at 100 °C and held for 2 min, then increased to 140 °C and held for 6 min. The VFA production was calculated by first converting the measured VFAs (C2-C6) concentrations (mg·L^−1^) to COD (mgCOD·L^−1^), then divided by the COD of the effluent and times 100%.

The protease activity was measured with the Pierce™ Fluorescent Protease Activity kit (Thermo Scientific, USA), including contents of FTC-Casein, TPCK Trypsin, and BupH™ Tris-buffered saline. Measurement was carried out in triplicates, 100 μL sample taken directly from the reactors was mixed with 100 μL FTC-Casein working reagent (10 μg·mL^−1^ FTC-Casein in 25 mM Tris, 0.15 M NaCl, pH 7.2 assay buffer) in a 96-well plate (Pierce White Opaque 96-Well Plates). The plate was incubated for 2 h at 25 °C inside the fluorometer (FLUOstar, galaxy), the fluorescence was read every 120 s, and the fluorescein filter was set between 485 nm excitation wavelength and 520 nm emission wavelength. The reads of each sample were converted to BAEE (fluorescence unit, Na-Vebzoyl-L-Arginine Ethyl Ester, units·L^−1^), based on the calibration curve built with reference protease, Trypsin, and the conversion factor between its concentration and BAEE (14,000 BAEE units·L^−1^ equivalent can be produced by 50 mg·L^−1^ Trypsin). Final protease activity was calculated by linear fitting of the BAEE measurements and then normalized with the COD concentration of the sample.

### Microbial community analysis

For microbial community analysis, samples were taken at the beginning and the end (stable operation) of each stage, as shown in Table 1. Aliquots of 2 mL liquid sample were centrifuged at 14,000 × g for 5 min, the supernatant was removed, and then the sample was stored at -20 °C before DNA extraction with FastDNA™ Spin Kit for Soil (MP BIOMEDICALS). The extracted DNA concentrations in all samples were checked by the Qubit 3 Fluorometer (Thermo Fisher Scientific, USA) to ensure a DNA concentration higher than 20 ng·μL^−1^. The DNA samples were then sent for amplicon sequencing by Illumina NovaSeq 6000 platform (Novogene, UK), using the primers 341F [(5′–3′) CCTAYGGGRBGCASCAG] and 806R [(5′–3′) GGACTACNNGGGTATCTAAT] for bacteria/archaea in the V3–V4 regions. Paired-end reads (2 × 250 bp) were assigned to samples based on their unique barcodes and truncated by cutting off the barcode and primer sequences. Paired-end reads were merged using FLASH (V1.2.7) (Magoč and Salzberg 2011), and the splicing sequences were called raw tags. High-quality clean tags (Bokulich et al. 2013) were obtained by quality filtering on the raw tags according to the QIIME (V1.7.0) (Caporaso et al. 2010). The clean tags were compared with the Gold database (see details http://drive5.com/uchime/uchime_download.html) using UCHIME algorithm (Edgar et al. 2011) to detect and remove chimera sequences (Haas et al. 2011) to obtain the effective tags. Sequences analysis were performed by Uparse (V7.0.1001) (Edgar 2013) using the effective tags. Sequences with ≥ 97% similarity were assigned to the same Operational Taxonomic Unit (OTU) and screened for further annotation by Mothur software against the SSUrRNA database of SILVA Database (Wang et al. 2007). Phylogenetic relationship of all OTUs was obtained by the MUSCLE (Version 3.8.31) (Edgar 2004). The sequences were deposited at DDBJ/EMBL/GenBank under the accession KFUH00000000.

**Table 1:** Microbiota samples taken at the beginning and end of each stage for microbial community analysis

		Stage I				Stage II		
		Inoculum	Day 62	Day 84		Day 112	Day 132	
CAS	Sample No	/	C1.2	C1.3		/	C2.2	C2.3
	Sample content		^a^Suspended microbiota	Suspended microbiota			Suspended microbiota	^b^Biofilm
LAC	Sample No	L1.1	L1.2	L1.3	L1.4	L2.1	L2.2	L2.3
	Sample content	Suspended microbiota	Suspended microbiota	Suspended microbiota	Biofilm	Suspended microbiota	Suspended microbiota	Biofilm

^a^Suspended microbiota was the suspended solids in the effluent;

^b^Biofilm sample was the microbiota attached to the inner wall of the reactor

/: Sample not valid due to low DNA concentration

OTUs abundance information was normalized using a standard of sequence number corresponding to the sample with the least sequences. Subsequent analysis of alpha diversity and beta diversity were all performed based on this output normalized data. Alpha diversity is applied in analysing biodiversity through observed species, Chao1, and phylogenetic diversity (PD). The indices in the samples were calculated with QIIME (V 1.7.0) and displayed with R software (V 2.15.3). Beta diversity analysis on weighted unifrac was calculated by QIIME (V 1.7.0). Principal Coordinate Analysis (PCoA) based on the weighted unifrac distance matrix of OTUs was performed and displayed by WGCNA package, stat packages and ggplot2 package in R software (V 2.15.3).

## Results

### COD balance and pH in the acidification reactors

**Fig. 2:**
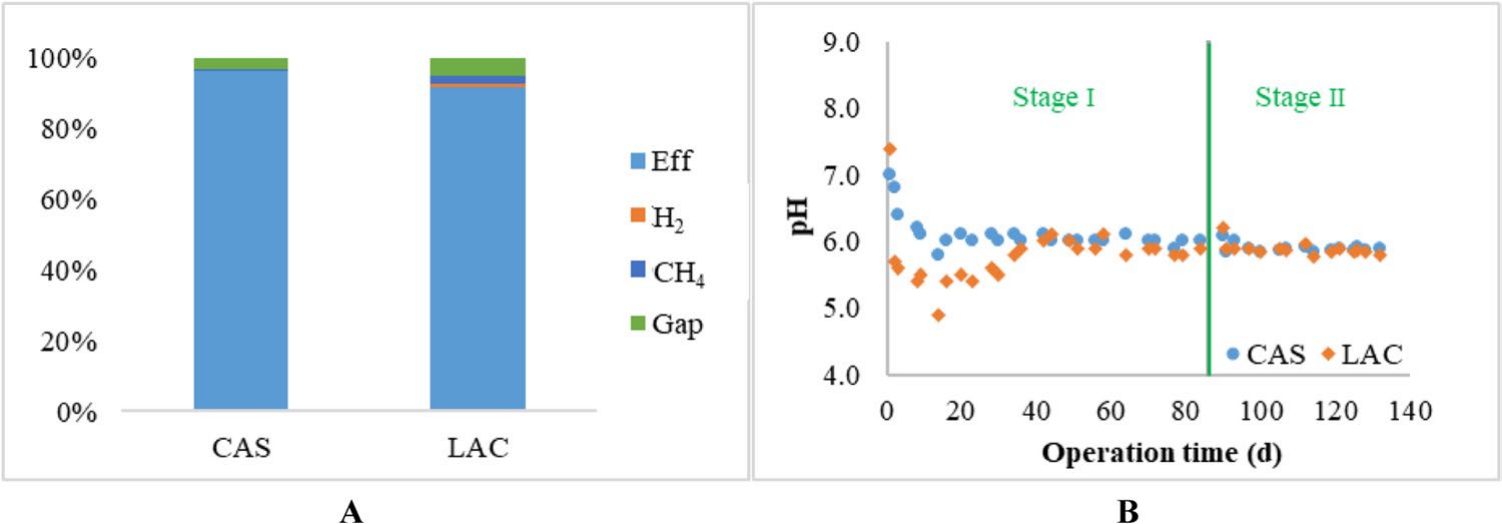
Average COD balance in the CAS and LAC reactors (**A**), where Eff, H_2_, and CH_4_ represent the output COD in the liquid effluent, and resulting biogas (H_2_ and CH_4_), the Gap represents the difference between the total output COD and input COD in the influent; pH in the two reactors recorded by the online pH probes (**B**)

The COD balance in the two acidification reactors during the operation period of 132 days is shown in Fig. 2A, the COD in the effluents was higher than 93% of the influent CODs of both the CAS and LAC reactors. The pH in the reactors was well regulated at 6 (Fig. 2B). Limited biogas gas production was expected at pH 6 (Holliger et al. 2016). Therefore, the COD in the two acidification reactors during the operation period was balanced with a maximum gap of 5%.

### Effect of the presence of lactose on deamination and VFA production

#### Deamination

**Fig. 3:**
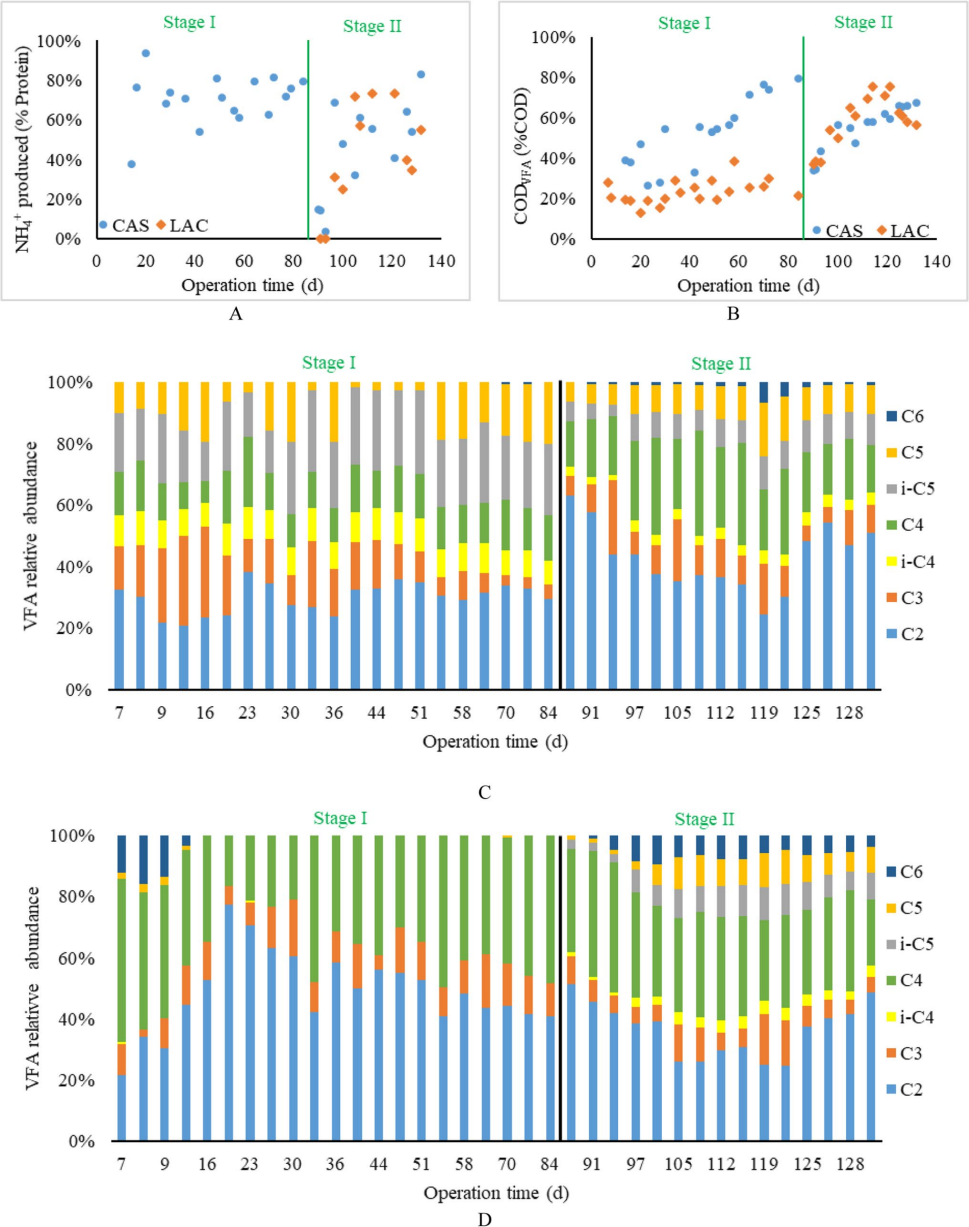
Deamination (**A**) and VFA production as % of effluent COD (**B**) in CAS reactor and LAC reactor during the operational period, VFA relative abundance in the CAS reactor (**C**), and VFA relative abundance in the LAC reactor (**D**)

Figure 3A shows the deamination efficiency of the two acidification reactors during the two stages. In stage I, casein was fed as the sole carbon and nitrogen source to the CAS reactor, and the deamination efficiency was calculated as the percentage of NH_4_^+^ released from the fed protein. The deamination efficiency increased from only 40% to almost 100% during the start-up period (day 0—20) and fluctuated between 60 and 80% during days 20—64. During days 70—84, a relatively stable period was observed, the average deamination degree was 77%, with a relative standard deviation of less than 5%. In the LAC reactor, lactose was fed as the carbon source and NH_4_^+^ was added as a nitrogen source, and therefore the deamination efficiency was 0%.

In stage II, both reactors were fed with the MIX (substrate C). The deamination efficiency in the CAS reactor dropped to 15% at the beginning of stage II. Subsequently, an increasing trend with fluctuations (between 41 and 64%) was observed, and a similar deamination efficiency as in stage I was achieved at the end of stage II (80%). Similarly, the deamination efficiency of the LAC reactor also increased from 4 to 73% during days 86—120 but decreased to 35—55% during days 120—132.

#### VFA production

Figure 3B shows that the effluent VFA content of the CAS reactor fluctuated between 30 and 55% of the effluent COD during days 0 – 49 (VFA concentration data can be found in supplementary material Table S1). It increased steadily from 51 to 64% of the effluent COD during days 49 – 64 and stabilized at 75 ± 5% during days 64 – 84, Whereas in the LAC reactor, the VFA production was relatively stable between 20 and 39% of the effluent COD throughout stage I, with an average of 23 ± 6%. The difference in VFA production efficiency between the CAS and LAC reactor was 40% in stage I.

In stage II, VFAs production in the CAS reactor initially decreased to 34% of the effluent COD, followed by a gradual increase to 67% during days 120—132. The variation in VFAs production agreed with the variation in deamination efficiency, that is: a sudden decrease in protein degradation at the beginning of stage II. The VFAs production in the LAC reactor considerably increased from 37 to 76% of the effluent COD during days 86 – 114; however, it started dropping to 60 ± 2% by the end of stage II. The observed increased COD to VFA conversion efficiency could be attributed to the production of the more reduced VFAs iC5, C5, and C6 (Fig. 3D) coinciding with protein degradation. The presence of lactose reduced the degradation efficiency of casein, resulting in a decreased deamination efficiency and VFAs production in the CAS reactor in stage II.

Figure 3C presents the VFA composition as the percentage of total VFA-COD in the CAS reactor during stage I and stage II. During days 56 – 84, the VFA production and composition were considered stable in stage I. The relative abundance of VFAs was similar to the fermentation products of gelatine at pH 6 in the study of Breure and van Andel (1984). When the substrate was changed to the MIX in stage II, the composition of VFAs during stable operation performance (days 125 – 132) had changed compared to that in stage I. The C2 increased from 30% ± 9% to 50 ± 3%, and C4 increased from 14 ± 3% to 23 ± 5%.

As shown in Fig. 3D, the fluctuations in VFA composition in the LAC reactor mainly concerned C2 and C4 during stage I. A relatively stable VFA composition was observed from day 64 to day 84, where the most abundant VFAs were C2 (43 ± 2%) and C4 (44 ± 4%). In stage II, the VFA composition in the LAC reactor gradually changed with the increasing production of iC4, iC5 and C5, and C6. This trend was also observed in the CAS reactor, and the identified main VFA products during stage II were C2 (42 ± 5%) and C4 (28 ± 5%).

### Effect of the presence of lactose on protease activity and deamination activity

#### Protease activity

**Fig. 4:**
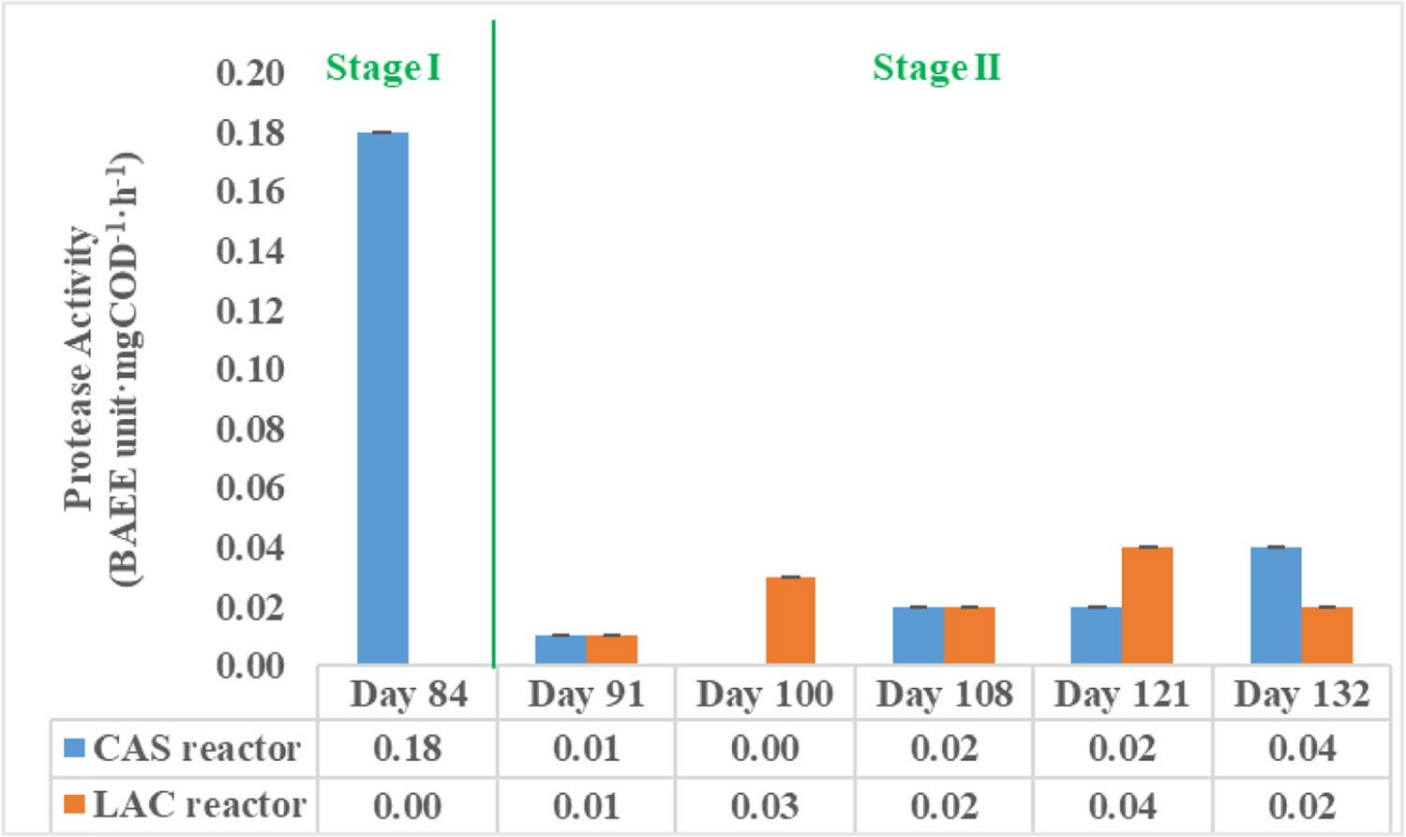
Protease activity measured in stage I and stage II (the column represents the average protease activity in BAEE unit·mgCOD^−1^·h.^−1^, error bars represent the standard deviation)

At the end of experimental stage I (day 84), the protease activity of the samples from both reactors was analysed. As shown in Fig. 4, protease activity of 0.18 ± 0.01 BAEE unit·mgCOD^−1^·h^−1^ was observed in the CAS reactor, whereas no protease activity was detected in the LAC reactor sample. During stage II (day 91 and day 132), the CAS and LAC reactors were both found to have protease activity but at a level lower than 0.04 ± 0.01 BAEE unit·mgCOD^−1^·h^−1^.

The results showed that with the presence of lactose, the protease activity decreased to lower than 25% of that in stage I in the CAS reactor, indicating a negative effect of lactose on the protease activity, whereas in the LAC reactor, protease activity was slowly developed under the presence of protein.

#### Deamination activity

**Fig. 5:**
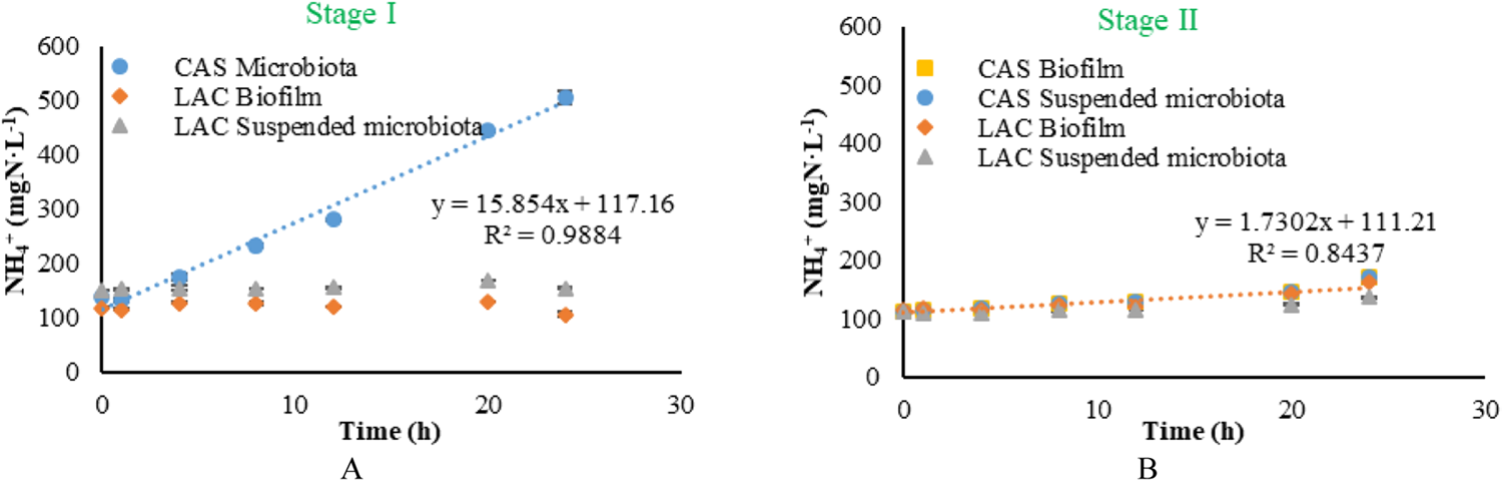
Ammonium production by microbiota sample taken at the end of stage I—84 days (**A**) and stage II – 132 days (**B**). The ammonium production rate was derived from the linear fitting of the plots; error bars represent the standard deviation

At the end of each stage, microbiota samples were taken from the reactor for assessing the deamination activity in batch tests. Figure 5A shows the ammonium production by the microbiota sample taken from the CAS and LAC reactors at the end of stage I. The deamination activity was calculated by linear fitting of the scattered plot of NH_4_^+^ concentrations as a function of time and divided by the microbiota concentration, 1.5 gVSS·L^−1^. Only suspended microbiota was found and collected from the CAS reactor (CAS microbiota) during this stage, whereas suspended microbiota (LAC suspended microbiota) and biofilm (LAC biofilm) were found and collected from the LAC reactor. The CAS microbiota had the highest deamination activity of 10.6 mg-N·gVSS^−1^·h^−1^. In contrast, no deamination activity of the LAC suspended microbiota and LAC biofilm was observed.

Figure 5B shows the ammonium production by the microbiota samples collected from both reactors at the end of stage II, including CAS suspended microbiota, CAS biofilm, LAC suspended microbiota and LAC biofilm. Compared to the deamination activity of CAS microbiota from stage I, the four microbiota samples from stage II showed a similarly low level of deamination activity, which was 1.2 mg-N·gVSS^−1^·h^−1^.

### Effect of the presence of lactose on microbial community

#### Microbial community composition in stage I and stage II

**Fig. 6:**
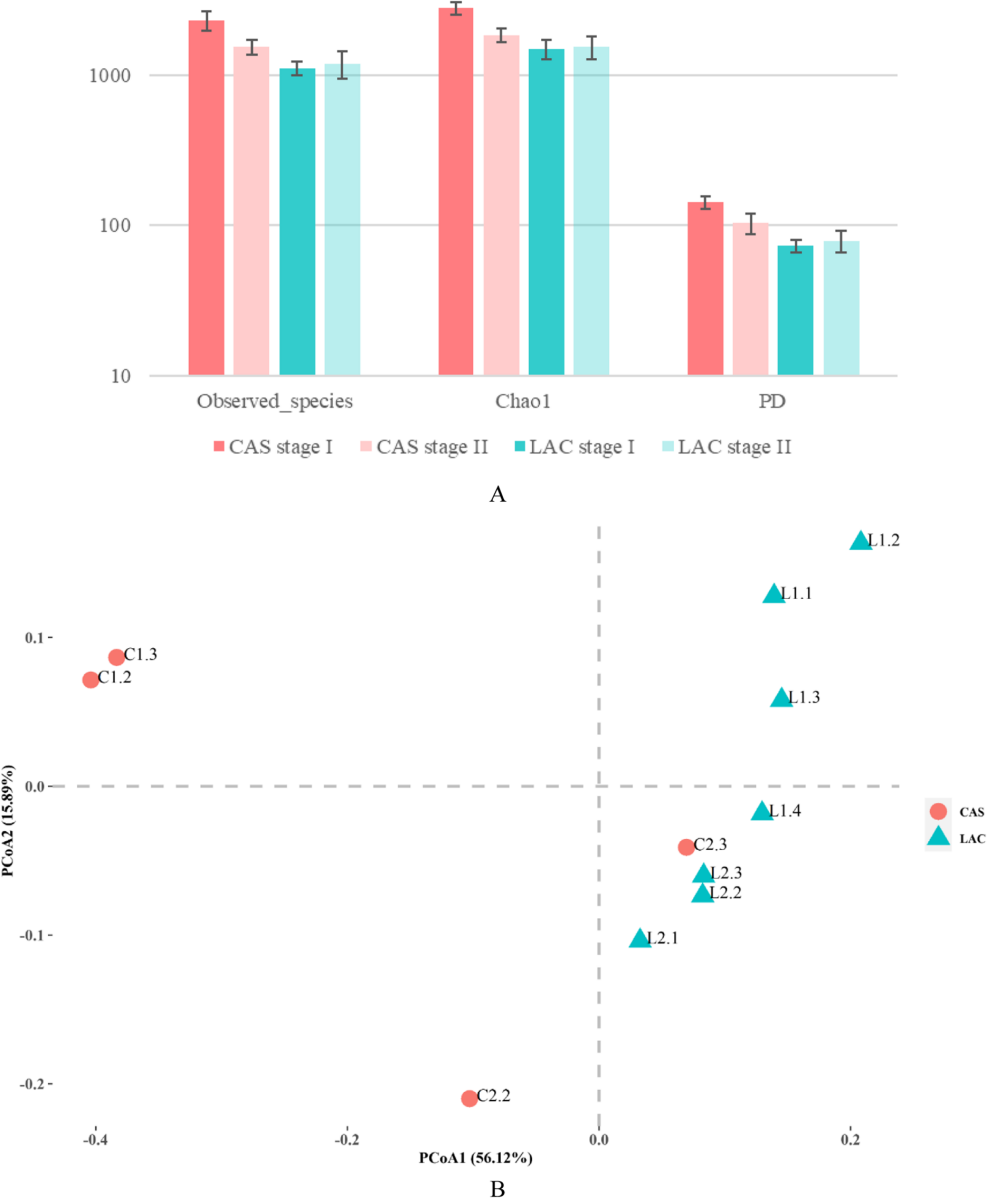

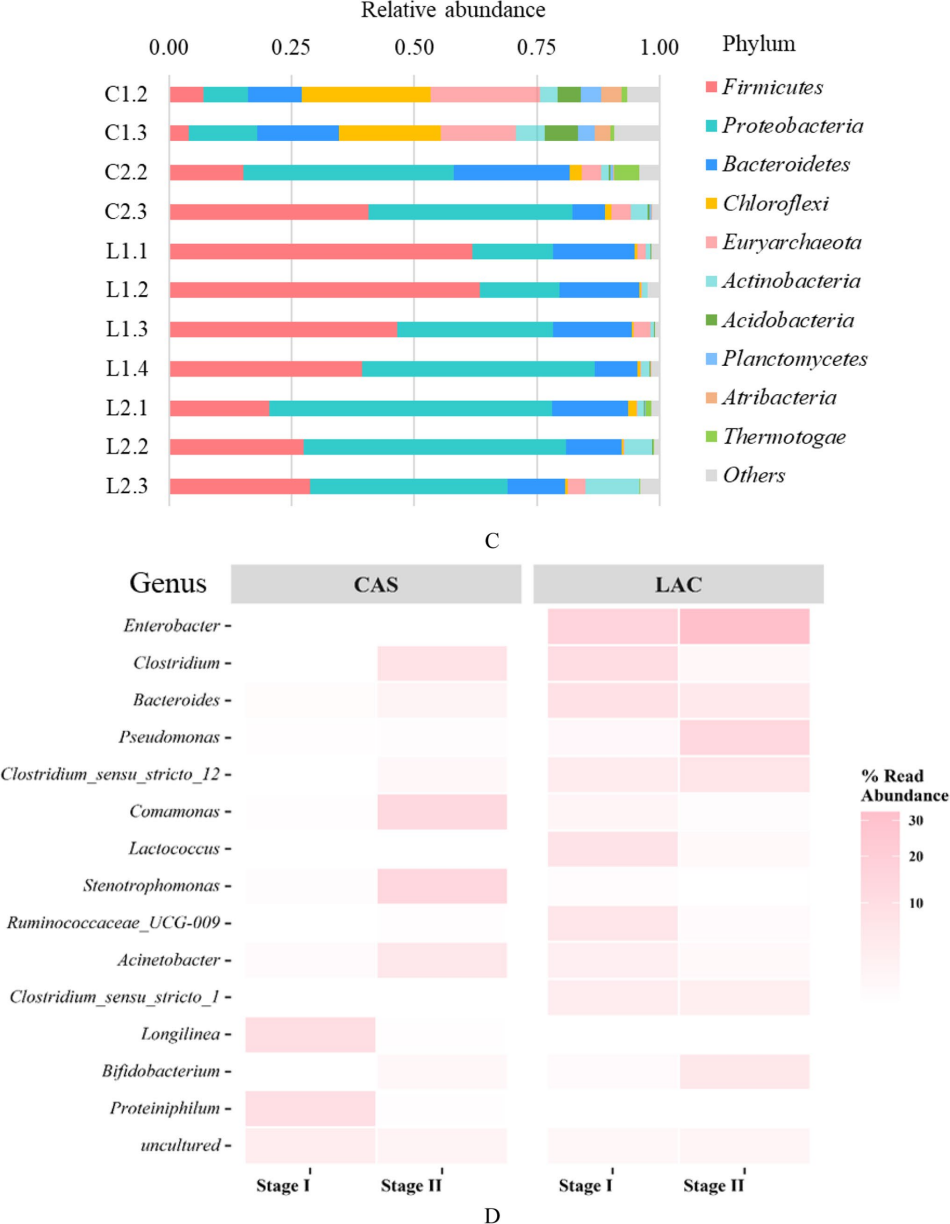
Alpha diversity of the microbial community in CAS and LAC reactors during stage I and stage II, average values of the observed species, Chao1, and phylogenetic diversity (PD) are presented and the error bars indicate the minimum and maximum values of the samples in each stage (**A**), Principal coordinates analysis (PCoA) ordination based on the weighted unifrac distance matrix of OTUs comparing all samples taken from CAS and LAC reactors during stage I and stage II. The PCoA is coloured by reactor: red symbols indicate CAS, the green ones indicate LAC. The percentage of the variation explained by the plotted principal coordinates is indicated on the axes (**B**), phylum relative abundance of microbiota in samples from CAS and LAC reactors during stage I and stage II (**C**), and top 15 dominant genera in CAS and LAC reactors during stage I and stage II (**D**)

Alpha diversity of the samples was compared using three indices, the observed species, chao1 and phylogenetic diversity indices. As can be seen in Fig. 6A, samples from the CAS reactor, especially during stage I, showed a higher diversity. In Fig. 6B, the PCoA analysis illustrated the similarity of the microbial communities in all samples that are indicated in Table 1. The distance between samples indicates their similarity in microbial community, the shorter the distance, the higher the similarity, and vice versa. As shown in the figure, samples C1.2 and C1.3 taken from the CAS reactor stage I, displayed the longest distance to the rest of the samples, indicating that the microbial community under casein-fed conditions had a very different composition than the lactose-fed or MIX-fed conditions. The samples L2.2 and L2.3, taken from the LAC reactor, displayed the shortest distance, indicating that the microbial community can be considered similar in the suspended microbiota and biofilm in this reactor by the end of stage II. Remarkably, suspended microbiota L1.3 has a shorter distance to stage I samples, whereas biofilm sample L1.4 was closer to stage II samples. This means that the microbial community composition in the biofilm samples of stage I became predominant in stage II in LAC reactor.

Figure 6C shows the relative abundance at phylum level in each sample. When comparing the difference between stage I and stage II in the CAS reactor, the phyla *Firmicutes* and *Proteobacteria* were promoted by the presence of lactose and increased from 5.5% and 11.5% OTUs (average of C1.2 and C1.3) to 41% and 42% OTUs (C2.3), respectively. At the same time, the phyla *Chloroflexi* and *Euryarchaeota* (Archaea) were predominant in stage I but decreased in relative abundance in stage II from 23.5% and 18.5% OTUs (average of C1.2 and C1.3) to 1% and 4% OTUs (C2.3), respectively. Microorganisms belonging to these phyla had a slower growth rate than the acid producing bacteria at pH 6. The phylum *Bacteroidetes* exhibited first an increase from 14% OTUs (average of C1.2 and C1.3) to 24% OTUs (C2.2) and then decreased to 7% OTUs (C2.3). With the feed changed from casein to the MIX, the composition of the microbial community in the CAS reactor significantly changed at the phylum level, and the presence of lactose promoted the growth of bacteria affiliated to phyla *Proteobacteria* and *Firmicutes*.

When comparing the differences in microbial community composition between stage I and stage II in the LAC reactor, the phylum *Firmicutes* was found to be predominant in stage I, accounting for more than 60% OTUs in L1.1 and L1.2, 47% OTUs in L1.3, and 39% OTUs in L1.4. However, in stage II, *Firmicutes* decreased to 29% OTUs (L2.3). The phylum *Proteobacteria* were the second most abundant in L1.1 and L1.2 (16% OTUs) and gradually became more abundant than *Firmicutes* in L1.4, with a relative abundance of 47% OTUs, and was maintained above 40% OTUs during stage II. Bacteria from the phylum *Bacteroidetes* was also abundantly present in all the LAC samples, it accounted for 17% OTUs in L1.1 and 12% OTUs in L2.3, and the composition of the microbial community in the LAC reactor remained relatively stable during the two different stages.

According to the alpha and beta diversity analysis, the microbiota, that was acclimated to casein as the sole substrate, was significantly different from the microbial community acclimated to lactose as the sole substrate. The phyla *Chloroflexi* and *Euryarchaeota* (Archaea) were predominant in the casein-fed reactor, whereas *Proteobacteria* and *Firmicutes* were predominant when lactose was present in the reactor. In general, the microbial community had a higher similarity in stage II, when lactose was present.

#### Characterization of predominant genera

Figure 6D presents the top 15 predominant genera in stage I and stage II in CAS and LAC reactors. In the CAS reactor, it was found that genera *Longilinea*, *Proteiniphilum*, and *midas_g_156* were predominant in stage I, whereas genera *Stenotrophomonas*, *Comamonas*, *Clostridium*, *Acinetobacter*, and *Bacteroides* were dominating in stage II. Among the dominating genera found in CAS reactor, the genera *Longilinea*, *Proteiniphilum*, *Stenotrophomonas*, *Acinetobacter*, and *Clostridium *sensu stricto are identified as facultative, i.e., being able to ferment both sugars and proteins/amino acids (see Table 2). Whereas genus *Comamonas* is identified as only able to ferment proteins/amino acids, genus *Bacteroides* is identified as only able to ferment sugars (Dueholm et al. 2021).

**Table 2:** Substrate-utilization of the identified top 33 predominant genera

Phylum	Genus	Substrate-utilization		Environment	Reference
		Sugar	Proteins/amino acids		
*Actinobacteria*	*Bifidobacterium*	n.a	n.a	^a^Stage II Mixture-feeding	(Dueholm et al. 2021)
		Y	n.a	Animal gut	(Milani et al. 2014)
*Atribacteria*	*Candidatus caldatribacterium*	n/a	n.a	Stage I Protein-feeding	(Dueholm et al. 2021)
		Y	n.a	Glucose (and chemicals)-feeding AD reactor	(Schwan et al. 2020)
*Bacteroidetes*	*Acetobacteroides*	Y	Y	Stage II Mixture-feeding	(Dueholm et al. 2021)
	*Bacteroides*	Y	N	Stage I Sugar-feeding	(Dueholm et al. 2021)
	*Elizabethkingia*	n.a	n.a	Stage I Sugar-feeding	(Dueholm et al. 2021)
	*Macellibacteroides*	n.a	n.a	Stage II Mixture-feeding	(Dueholm et al. 2021)
		Y	Y	Slaughterhouse wastewater-feeding UFMB reactor	(Jabari et al. 2012)
	*Petrimonas*	n.a	n.a	Stage II Mixture-feeding	(Dueholm et al. 2021)
		Y	N		(Hahnke et al. 2016)
	*Prevotella (9)*	Y	Y	Stage II Mixture-feeding	(Dueholm et al. 2021)
	*Proteiniphilum*	Y	Y	Stage I Protein-feeding	(Dueholm et al. 2021)
	*Sphingobacterium*	n.a	n.a	Stage I Sugar-feeding	(Dueholm et al. 2021)
		Y	Y	Human gut	(Yabuuchi et al. 1983)
*Chloroflexi*	*Longilinea*	Y	Y	Stage I Protein-feeding	(Dueholm et al. 2021)
*Firmicutes*	*Acetoanaerobium*	n.a	n.a	Stage II Mixture-feeding	(Dueholm et al. 2021)
		Y	Y	oil exploration drilling site; Pulp and paper mill-feeding AD reactor	(Duan et al. 2016; Sleat et al. 1985)
	*Blautia*	n.a	n.a	Stage I Sugar-feeding	(Dueholm et al. 2021)
		Y	n.a	Human gut	(Maturana and Cárdenas 2021)
	*Caproiciproducens*	n.a	n.a	Stage I Sugar-feeding; Stage II Mixture-feeding	(Dueholm et al. 2021)
		Y	Y	Galactitolivorans wastewater treatment plant	(Kim et al. 2015)
	*Clostridium *sensu stricto* 1*	Y	Y	Stage I Sugar-feeding; Stage II Mixture-feeding	(Dueholm et al. 2021)
	*Clostridium *sensu stricto* 2*	n.a	n.a	Stage I Sugar-feeding; Stage II Mixture-feeding	(Dueholm et al. 2021)
		Y	Y		(Udaondo et al. 2017)
	*Clostridium *sensu stricto* 9*	n.a	n.a	Stage II Mixture-feeding	(Dueholm et al. 2021)
		Y	Y		(Udaondo et al. 2017)
	*Clostridium *sensu stricto* 12*	Y	Y	Stage I Sugar-feeding; Stage II Mixture-feeding	(Dueholm et al. 2021)
	*Erysipelotrichaceae (UCG-004)*	n.a	n.a	Stage II Mixture-feeding	(Dueholm et al. 2021)
		Y	n.a	Jersey cows diet-feeding	(Deusch et al. 2017)
	*Lactococcus*	Y	Y	Stage I Sugar-feeding	(Dueholm et al. 2021)
	*Lachnoclostridium (5)*	Y	N	Stage I Sugar-feeding; Stage II Mixture-feeding	(Dueholm et al. 2021)
	*Lactobacillus*	n.a	n.a	Stage II Mixture-feeding	(Dueholm et al. 2021)
		Y	n.a	Fermented food production	(Salvetti et al. 2012)
	*Pygmaiobacter*	n.a	n.a	Stage I Sugar-feeding	(Dueholm et al. 2021)
		N	Y	Human gut	(Bilen et al. 2016)
	*Ruminococcaceae (UCG-009)*	n.a	n.a	Stage I Sugar-feeding	(Dueholm et al. 2021)
		Y	n.a	animal gut	(Bäckhed et al. 2004)
*Proteobacteria*	*Acinetobacter*	Y	Y	Stage II Mixture-feeding	(Dueholm et al. 2021)
		N	Y	Human microbiome	(Bouvet and Jeanjean 1989)
	*Comamonas*	N	Y	Stage II Mixture-feeding	(Dueholm et al. 2021)
	*Delftia*	n.a	n.a	Stage II Mixture-feeding	(Dueholm et al. 2021)
		n.a	Y		(Braña et al. 2016)
	*Hydrogenophaga*	n.a	n.a	Stage I Sugar-feeding	(Dueholm et al. 2021)
		Y	N		(Willems et al. 1989)
	*Parasutterella*	n.a	n.a	Stage II Mixture-feeding	(Dueholm et al. 2021)
		Y	n.a	Human and mouse gut	(Ju et al. 2019)
	*Pseudomonas*	Y	Y	Stage II Mixture-feeding	(Dueholm et al. 2021)
	*Stenotrophomonas*	n.a	n.a	Stage II Mixture-feeding	(Dueholm et al. 2021)
*Synergistetes*	*JGI-0000079-D21*	n.a	n.a	Stage I Protein-feeding	(Dueholm et al. 2021)
		N	Y	Terephthalate degrading reactor	(Chen et al. 2020)
*Thermotogae*	*Mesotoga*	n.a	n.a	Stage II Mixture-feeding	(Dueholm et al. 2021)
		Y	Y		(Nesbø et al. 2019)

^a^Stage I and Stage II refers to this study;

n.a.: not available;

Y: yes;

N: no

During stage I of the LAC reactor, the dominating genera included *Enterobacter*, *Clostridium*, *Bacteroides*, *Lactococcus*, *Ruminococcaceae UCG 009*, *Acinetobacter*, *Clostridium *sensu stricto* 1*, and *Clostridium *sensu stricto* 12*. The dominating genera in stage II were *Enterobacter*, *Pseudomonas*, *Clostridium *sensu stricto* 12*, *Bacteroides*, *Bifidobacterium*, and *Clostridium *sensu stricto* 1*. As shown in Table 2, in addition to the genera mentioned in the previous paragraph, genera *Proteiniphilum*, *Pseudomonas*, *Ruminococcaceae UCG 009*, and *Bifidobacterium* are reported to be only able to ferment sugars. Whereas *Lactococcus* is identified as a facultative genus, which can ferment both proteins/amino acids and sugars.

**Fig. 7:**
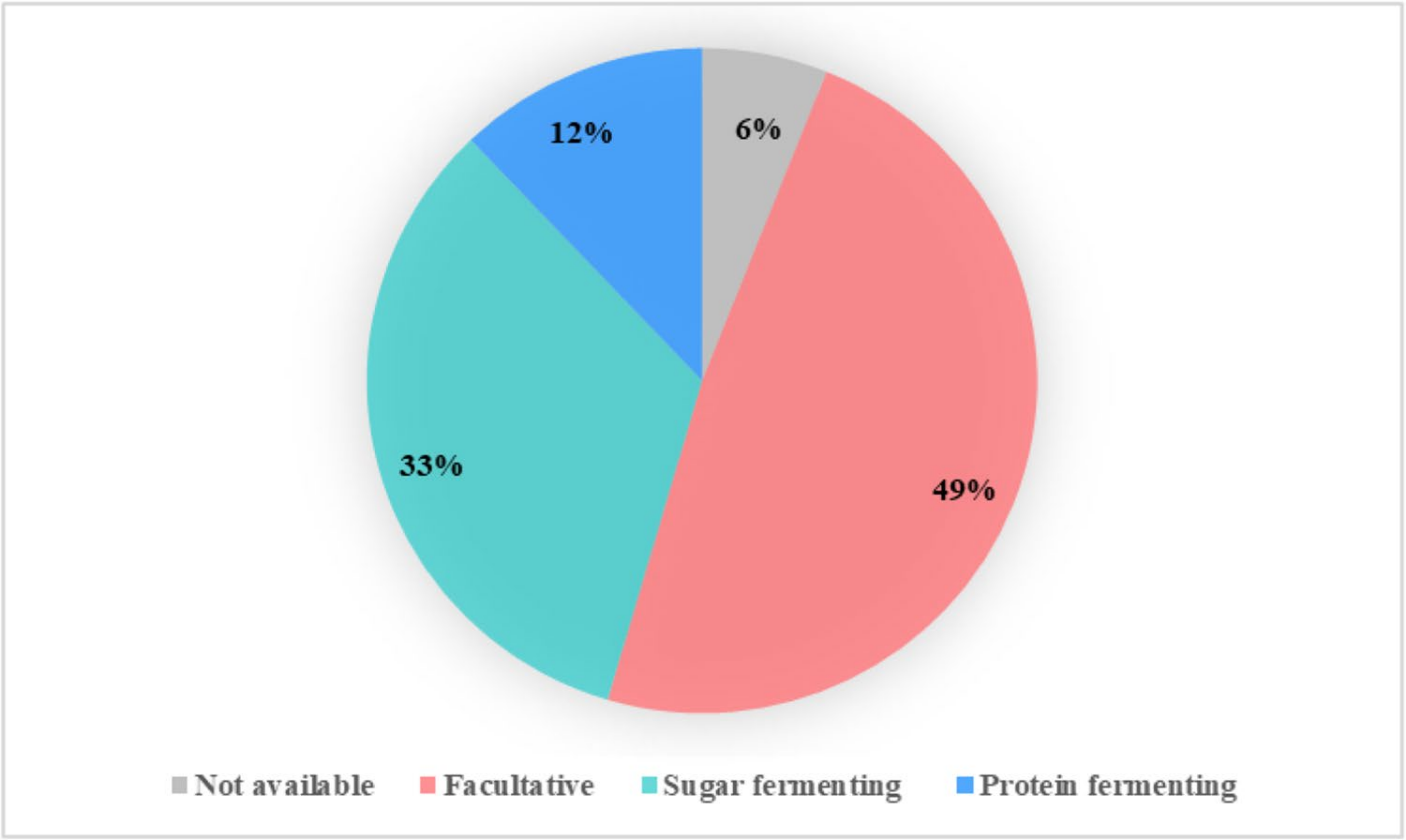
Categorization of the top 33 genera in all samples according to substrate utilization characteristics, facultative indicates the percentage of genera that can ferment both proteins and sugars, sugar fermenting indicates the percentage of genera that can only ferment sugars, protein fermenting indicates the percentage of genera that can only ferment proteins, and not available indicates the percentage of genera that were not able to characterized

Results in Table 2 show the 33 dominating genera identified in the collected samples, which has a relative abundance higher than 0.1% in at least one sample, and their ability to ferment sugars and proteins/amino acids is also presented. According to the abundance and substrate utilization information, the top 33 genera from all samples were categorized into 4 groups: 1) facultative bacteria, able to ferment both sugars and proteins; 2) sugar fermenting bacteria, able to ferment sugars but not proteins; 3) protein fermenting bacteria, able to ferment proteins but not sugars; and 4) not available, substrate utilization information was not available. We calculated the percentage of each group in the top 33 genera by counting the number of genera in each group and divided it by the total number of genera (33), and plotted the results as shown in Fig. 7. Half of the identified genera (49%) are reported to be facultative. Due to the limited availability of systematic genome analysis of some genera, the ability, to ferment proteins/amino acids, of some sugar-fermenting genera could not be fully confirmed, e.g. *Bifidobacterium* and *Ruminococcaceae UCG 009*, meaning that they could possibly be assigned to the facultative category as well.

## Discussion

As reported in previous studies, the presence of carbohydrates retards the production of protease, limits protein solubility and hydrolysis, and restricts the acidification of amino acids (Breure et al. 1986b; Glenn 1976; Yang et al. 2015). In this study, it was found that when the carbon source was changed from casein to the mixture of casein and lactose, the efficiency of deamination in the protein-fed reactor decreased from 77 to 15% and the VFA production decreased from 75 to 34% of the effluent COD (Fig. 3).

Additionally, a decrease in protease activity and deamination activity of the microbiota was also observed in the presence of lactose (Figs. 4 and 5), which has not been reported before. The deamination activity was 10.6 mg-N·gVSS^−1^·h^−1^ when the microbiota was acclimated to pure casein, it decreased to about 10% after the microbiota was acclimated to the MIX, and it took at least a month to acclimate the microbial community to the MIX to achieve a similar deamination efficiency as in stage I. Although the protein concentration in stage II was half that in stage I, it was not the reason for the lower deamination efficiency, as casein is completely deaminated within 20 h at both 6.0 g·L^−1^ and 3.0 g·L^−1^ (Deng et al. 2022). In the presence of lactose, the microbial community has a low protease activity and deamination activity, and only degraded casein to a limited extent. These results are in agreement with the study carried out by Breure et al. (1986a), which shows that gelatine degradation is retarded when glucose is present as a second carbon source.

Although the retardation of protein degradation by the presence of carbohydrates was frequently observed, the mechanism of the phenomenon has not been revealed. Our study investigated the effect of lactose on the microbial community and found that when lactose was present, the microbial community, previously acclimated to casein, became similar to the microbial community acclimated to lactose (Fig. 6A). The results indicated a selection of microorganisms by the presence of lactose. Generally, *Firmicutes* are the most abundant under various environmental conditions in anaerobic reactors (Pasalari et al. 2021), *Chloroflexi* and *Firmicutes* are dominant in protein-fed reactors (Kovács et al. 2013; Palatsi et al. 2011). In our study, the phyla *Chloroflexi, Proteobacteria*, and *Bacteroidetes* were predominant in the casein-fed reactor, whereas *Proteobacteria* and *Firmicutes* were predominant when lactose was present in the reactor. The observed retardation of protein degradation can be correlated to the shift in the microbial community in the presence of lactose.

Furthermore, among the identified microorganisms, genera that can ferment both proteins/amino acids and carbohydrates were the predominant population in the microbial community (Table 2). Thermodynamically, sugar fermentation yields more energy than proteins/amino acids: cells gain 1–2 mol ATP·mol^−1^ glucose (Zhou et al. 2018) compared to 0.5 mol ATP·mol^−1^ amino acids (Barker 1981). The bacteria that can ferment sugars, including the facultative bacteria and the sugar fermenters, have a higher growth rate and biomass yield than the bacteria that are only fermenting proteins/amino acids (Pavlostathis and Giraldo-Gomez 1991; Tang et al. 2005), and it is, therefore, expected that the former can outgrow the latter during the mixture-feeding conditions at a specified solids retention time. Consequently, lactose was degraded prior to casein.

To confirm that the predominant facultative microorganisms are the active protein degraders, further research on the functional analysis of the microbial community is recommended. In addition, microbial community analysis has been focused on the methane-producing archaea and the granulation-involving filamentous (De Vrieze and Verstraete 2016), and less attention has been given to the protein degraders in a complex microbiota. To better manage and control the AD process for the treatment of protein-rich streams, an in-depth understanding of the protein degraders in complex microbiota is required.

In conclusion, the retardation of casein degradation by the presence of lactose was due to 1) the substrate-preference of the dominant bacteria, including the facultative bacteria and the sugar fermenters, and 2) the higher growth rate and yield of the dominant bacteria. Overall, this study offers a better understanding of the mechanisms behind the retardation of protein degradation by the presence of carbohydrates, which can provide insights into the design of the anaerobic digestion process of protein-rich waste streams to improve protein degradation.

## Supplementary Information

Below is the link to the electronic supplementary material.

Supplementary file1 (PDF 22 KB)

## Data Availability

All data generated or analysed during this study are included in this manuscript and supplementary material.

## References

[CR1] Adhikari BB, Chae M, Bressler DC (2018) Utilization of Slaughterhouse Waste in Value-Added Applications: Recent Advances in the Development of Wood Adhesives. Polymers (Basel) 10(2) 10.3390/polym10020176 10.3390/polym10020176 PMC6415179 30966212

[CR2] APHA (1998) American Public Health Association/American Water works/Water Environment Federation, Washington DC

[CR3] Bäckhed F, Ding H, Wang T, Hooper LV, Koh GY, Nagy A, Semenkovich CF, Gordon JI (2004). The gut microbiota as an environmental factor that regulates fat storage. PNAS.

[CR4] Baker BR, Mohamed R, Al-Gheethi A, Aziz HA (2021). Advanced technologies for poultry slaughterhouse wastewater treatment: A systematic review. J Dispers Sci Technol.

[CR5] Barker HA (1961) Chapter 3 - Fermentations of nitrogenous organic compounds. Metab Clin Exp 151–207 10.1016/B978-0-12-395627-9.50011-6

[CR6] Barker HA (1981). (1981) Amino Acid Degradation By Anaerobic Bacteria. Ann Rev Biochem.

[CR7] Bilen M, Mbogning MD, Cadoret F, Dubourg G, Daoud Z, Fournier PE, Raoult D (2016) “*Pygmaiobacter massiliensis*” sp., nov., a new bacterium isolated from the human gut of a pygmy female. NMNI 10.1016/j.nmni.2016.12.015 10.1016/j.nmni.2016.12.015 PMC5284491 28179983

[CR8] Bokulich NA, Subramanian S, Faith JJ, Gevers D, Gordon JI, Knight R, Mills DA, Caporaso JG (2013). Quality-filtering vastly improves diversity estimates from Illumina amplicon sequencing. Nat Methods.

[CR9] Bouvet P, Jeanjean S (1989). Delineation of new proteolytic genomic species in the genus *Acinetobacter*. Res Microbiol.

[CR10] Braguglia CM, Gallipoli A, Gianico A, Pagliaccia P (2018). Anaerobic bioconversion of food waste into energy: A critical review. Bioresour Technol.

[CR11] Braña V, Cagide C, Morel MA (2016) Microbial Models: From Environmental to Industrial Sustainability. Castro-Sowinski S (ed), pp. 227–247, Springer, Singapore

[CR12] Breure AM, Beeftink HH, Verkuijlen J, van Andel JG (1986). Acidogenic fermentation of protein/carbohydrate mixtures by bacterial populations adapted to one of the substrates in anaerobic chemostat cultures. Appl Microbiol Biotechnol.

[CR13] Breure AM, Mooijman KA, van Andel JG (1986). Protein degradation in anaerobic digestion: influence of volatile fatty acids and carbohydrates on hydrolysis and acidogenic fermentation of gelatin. Appl Microbiol Biotechnol.

[CR14] Breure AM, van Andel JG (1984). Hydrolysis and acidogenic fermentation of a protein, gelatin, in an anaerobic continuous culture. Appl Microbiol Biotechnol.

[CR15] Bustillo-Lecompte CF, Mehrvar M (2015). Slaughterhouse wastewater characteristics, treatment, and management in the meat processing industry: A review on trends and advances. J Environ Manage.

[CR16] Caporaso JG, Kuczynski J, Stombaugh J, Bittinger K, Bushman FD, Costello EK, Fierer N, Peña AG, Goodrich JK, Gordon JI (2010). QIIME allows analysis of high-throughput community sequencing data. Nat Methods.

[CR17] Chen I-MA, Chu, K, Palaniappan K, Ratner A, Huang J, Huntemann M, Hajek P, Ritter S, Varghese N, Seshadri R, Roux S, Woyke T, Eloe-Fadrosh EA, Ivanova NN, Kyrpides Nikos C (2020) The IMG/M data management and analysis system v.6.0: new tools and advanced capabilities. Nucleic Acids Res 49(D1):D751-D763 10.1093/nar/gkaa939 10.1093/nar/gkaa939 PMC7778900 33119741

[CR18] De Vrieze J, Verstraete W (2016). Perspectives for microbial community composition in anaerobic digestion: from abundance and activity to connectivity. Environ Microbiol.

[CR19] Deng Z, Ferreira ALM, van Lier JB, Spanjers H (2022) Kinetic study on the effects of protein structure complexity, protein concentrations, carbohydrates, and VFAs on anaerobic protein degradation, Under review. Department of Water Management, Delft University of Technology.

[CR20] Deusch S, Camarinha-Silva A, Conrad J, Beifuss U, Rodehutscord M, Seifert J (2017). A structural and functional elucidation of the rumen microbiome influenced by various diets and microenvironments. Front Microbiol.

[CR21] Duan J, Huo X, Du W, Liang J, Wang D, Yang S (2016) Biodegradation of kraft lignin by a newly isolated anaerobic bacterial strain, *Acetoanaerobium* sp. WJDL-Y2. Lett Appl Microbiol 62(1):55–62 10.1111/lam.12508 10.1111/lam.12508 26465801

[CR22] Dueholm MKD, Nierychlo M, Andersen KS, Rudkjøbing V, Knutsson S, Albertsen M, Nielsen PH, 2022. MiDAS 4: A global catalogue of full-length 16S rRNA gene sequences and taxonomy for studies of bacterial communities in wastewater treatment plants. Nat Commun 13:1908. 10.1038/s41467-022-29438-7 PMC8989995 35393411

[CR23] Edgar RC (2004). MUSCLE: multiple sequence alignment with high accuracy and high throughput. Nuleic Acids Res.

[CR24] Edgar RC (2013). UPARSE: highly accurate OTU sequences from microbial amplicon reads. Nat Methods.

[CR25] Edgar RC, Haas BJ, Clemente JC, Quince C, Knight R (2011). UCHIME improves sensitivity and speed of chimera detection. Bioinformatics.

[CR26] Ganesh Saratale R, Kumar G, Banu R, Xia A, Periyasamy S, DattatrayaSaratale G (2018). A critical review on anaerobic digestion of microalgae and macroalgae and co-digestion of biomass for enhanced methane generation. Bioresour Technol.

[CR27] Glenn AR (1976). Production of extracellular proteins by bacteria. Ann Rev Microbiol.

[CR28] Haas BJ, Gevers D, Earl AM, Feldgarden M, Ward DV, Giannoukos G, Ciulla D, Tabbaa D, Highlander SK, Sodergren E (2011). Chimeric 16S rRNA sequence formation and detection in Sanger and 454-pyrosequenced PCR amplicons. Genome Res.

[CR29] Hahnke S, Langer T, Koeck DE, Klocke M (2016) Description of *Proteiniphilum saccharofermentans* sp. nov., *Petrimonas mucosa* sp. nov. and *Fermentimonas caenicola* gen. nov., sp. nov., isolated from mesophilic laboratory-scale biogas reactors, and emended description of the genus *Proteiniphilum*. Int J Syst Evol Microbiol 66(3):1466–1475 10.1099/ijsem.0.000902 10.1099/ijsem.0.000902 26782749

[CR30] Hassan A, Nelson B (2012). Invited review: anaerobic fermentation of dairy food wastewater. J Dairy Sci.

[CR31] Hendriks A, van Lier JB, de Kreuk MK (2018). Growth media in anaerobic fermentative processes: The underestimated potential of thermophilic fermentation and anaerobic digestion. Biotechnol Adv.

[CR32] Holliger C, Alves M, Andrade D, Angelidaki I, Astals S, Baier U, Bougrier C, Buffiere P, Carballa M, de Wilde V, Ebertseder F, Fernandez B, Ficara E, Fotidis I, Frigon JC, de Laclos HF, Ghasimi DS, Hack G, Hartel M, Heerenklage J, Horvath IS, Jenicek P, Koch K, Krautwald J, Lizasoain J, Liu J, Mosberger L, Nistor M, Oechsner H, Oliveira JV, Paterson M, Pauss A, Pommier S, Porqueddu I, Raposo F, Ribeiro T, RuschPfund F, Stromberg S, Torrijos M, van Eekert M, van Lier J, Wedwitschka H, Wierinck I (2016). Towards a standardization of biomethane potential tests. Water Sci Technol.

[CR33] Jabari L, Gannoun H, Cayol J-L, Hedi A, Sakamoto M, Falsen E, Ohkuma M, Hamdi M, Fauque G, Ollivier B (2012) *Macellibacteroides fermentans* gen. nov., sp. nov., a member of the family *Porphyromonadaceae* isolated from an upflow anaerobic filter treating abattoir wastewaters. Int J Syst Evol Microbiol 62(Pt_10):2522–2527 10.1099/ijs.0.032508-0 10.1099/ijs.0.032508-0 22180609

[CR34] Ju T, Kong JY, Stothard P, Willing BP (2019). Defining the role of *Parasutterella*, a previously uncharacterized member of the core gut microbiota. ISME J.

[CR35] Kim B-C, Jeon BS, Kim S, Kim H, Um Y, Sang B-I (2015) *Caproiciproducens galactitolivorans* gen. nov., sp. nov., a bacterium capable of producing caproic acid from galactitol, isolated from a wastewater treatment plant. Int J Syst Evol Microbiol 65(Pt_12):4902–4908 10.1099/ijsem.0.000665 10.1099/ijsem.0.000665 26474980

[CR36] Kovács E, Wirth R, Maróti G, Bagi Z, Rákhely G, Kovács KL (2013). Biogas Production from Protein-Rich Biomass: Fed-Batch Anaerobic Fermentation of Casein and of Pig Blood and Associated Changes in Microbial Community Composition. PLoS One.

[CR37] Magdalena JA, Ballesteros M, Gonz C (2018). Efficient Anaerobic Digestion of Microalgae Biomass : Proteins as a Key Macromolecule. Molecules.

[CR38] Magoč T, Salzberg SL (2011). FLASH: fast length adjustment of short reads to improve genome assemblies. Bioinformatics.

[CR39] Mata-Alvarez J, Dosta J, Romero-Güiza MS, Fonoll X, Peces M, Astals S (2014). A critical review on anaerobic co-digestion achievements between 2010 and 2013. Renew Sust Energ Rev.

[CR40] Maturana JL, Cárdenas JP (2021) Insights on the evolutionary genomics of the *Blautia* genus: potential new species and genetic content among lineages. Front Microbiol 12. 10.3389/fmicb.2021.660920 10.3389/fmicb.2021.660920 PMC8107234 33981291

[CR41] Milani C, Lugli GA, Duranti S, Turroni F, Bottacini F, Mangifesta M, Sanchez B, Viappiani A, Mancabelli L, Taminiau B (2014). Genomic encyclopedia of type strains of the genus *Bifidobacterium*. Appl Environ Microbiol.

[CR42] Nesbø CL, Charchuk R, Pollo SM, Budwill K, Kublanov IV, Haverkamp TH, Foght J (2019) Genomic analysis of the mesophilic *Thermotogae* genus *Mesotoga* reveals phylogeographic structure and genomic determinants of its distinct metabolism. Environ Microbiol 21(1):456–470. 10.111/1462-2920.14477 30452102

[CR43] Palatsi J, Viñas M, Guivernau M, Fernandez B, Flotats X (2011). Anaerobic digestion of slaughterhouse waste: Main process limitations and microbial community interactions. Bioresour Technol.

[CR44] Pasalari H, Gholami M, Rezaee A, Esrafili A, Farzadkia M (2021). Perspectives on microbial community in anaerobic digestion with emphasis on environmental parameters: A systematic review. Chemosphere.

[CR45] Pavlostathis SG, Giraldo-Gomez E (1991). Kinetics of anaerobic treatment. Water Sci Technol.

[CR46] Salvetti E, Torriani S, Felis GE (2012). The genus *Lactobacillus*: a taxonomic update. Probiotics Antimicrob Proteins.

[CR47] Sanders WTM (2001). Anaerobic hydrolysis during digestion of complex substrates.

[CR48] Schwan B, Abendroth C, Latorre-Pérez A, Porcar M, Vilanova C, Dornack C (2020). Chemically stressed bacterial communities in anaerobic digesters exhibit resilience and ecological flexibility. Front Microbiol.

[CR49] Sleat R, Mah RA, Robinson R (1985) *Acetoanaerobium noterae* gen. nov., sp. nov.: an anaerobic bacterium that forms acetate from H_2_ and CO_2_. Int J Syst Evol Microbiol 35(1):10–15 10.1099/00207713-35-1-10

[CR50] Stewart EJ (2012). Growing unculturable bacteria. J Bacteriol.

[CR51] Tang Y, Shigematsu T, Morimura S, Kida K (2005). Microbial Community Analysis of Mesophilic Anaerobic Protein Degradation Process Using Bovine Serum Albumin ( BSA ) -Fed Continuous Cultivation. J Biosci Bioeng.

[CR52] Udaondo Z, Duque E, Ramos JL (2017). The pangenome of the genus *Clostridium*. Environ Microbiol.

[CR53] Wang LK, Hung Y-T, Lo HH, Yapijakis C (2005). Waste treatment in the food processing industry.

[CR54] Wang Q, Carrity GM, Tiedje JM, Cole JR (2007). Naïve Bayesian Classifier for Rapid Assignment of rRNA Sequences into the New Bacterial Taxonomy. Appl Environ Microbiol.

[CR55] Wang X, Lu X, Li F, Yang G (2014). Effects of temperature and Carbon-Nitrogen (C/N) ratio on the performance of anaerobic co-digestion of dairy manure, chicken manure and rice straw: Focusing on ammonia inhibition. PLoS One.

[CR56] Willems A, Busse J, Goor M, Pot B, Falsen E, Jantzen E, Hoste B, Gillis M, Kersters K, Auling G (1989) *Hydrogenophaga*, a new genus of hydrogen-oxidizing bacteria that includes *Hydrogenophaga flava* comb. nov.(formerly *Pseudomonas flava*), *Hydrogenophaga palleronii* (formerly *Pseudomonas palleronii*), *Hydrogenophaga pseudoflava* (formerly *Pseudomonas pseudoflava* and “*Pseudomonas carboxydoflava*”), and *Hydrogenophaga taeniospiralis* (formerly *Pseudomonas taeniospiralis*). Int J Syst Evol Microbiol 39(3):319–333 10.1099/00207713-39-3-319

[CR57] Yabuuchi E, Kaneko T, Yano I, Wayne Moss C, MiyoshiI N (1983) *Sphingobacterium* gen. nov., *Sphingobacterium spiritivorum* comb. nov., *Sphingobacterium multivorum* comb. nov., *Sphingobacterium mizutae* sp. nov., and *Flavobacterium indologenes* sp. nov.: glucose-nonfermenting gram-negative rods in CDC groups IIK-2 and IIb. Int J Syst Evol Microbiol 33(3):580–598 10.1099/00207713-33-3-580

[CR58] Yang G, Zhang P, Zhang G, Wang Y, Yang A (2015). Degradation properties of protein and carbohydrate during sludge anaerobic digestion. Bioresour Technol.

[CR59] Yu H, Fang HHP (2001). Acidification of mid-and high-strength dairy wastewaters. Water Res.

[CR60] Yu H, Fang HHP (2003). Acidogenesis of gelatin-rich wastewater in an upflow anaerobic reactor: influence of pH and temperature. Water Res.

[CR61] Zhou M, Yan B, Wong JWC, Zhang Y (2018). Enhanced volatile fatty acids production from anaerobic fermentation of food waste: A mini-review focusing on acidogenic metabolic pathways. Bioresour Technol.

