# Supplementary material for: Characterization of microbial communities in anaerobic acidification reactors fed with casein and/or lactose
Source: Appl Microbiol Biotechnol. 2022 Aug 26;106(18):6301–16. doi: 10.1007/s00253-022-12132-5 (PMC9468126; doi:10.1007/s00253-022-12132-5)
Supplement: Supplementary file 1 — Supplementary file1 (PDF 22 KB) [file 253_2022_12132_MOESM1_ESM.pdf]

Applied Microbiology and Biotechnology

Ms. No. AMAB-D-22-00649R1

Characterization of microbial communities in anaerobic acidification reactors fed with casein and/or lactose

Authors

*Zhe Deng[a, b]

Ana Lucia Morgado Ferreira[b]

Henri Spanjers[a] (ORCID: 0000000290359417)

Jules B. van Lier[a] (ORCID: 0000000326075425)

*corresponding author

E: Z.Deng-2@tudelft.nl

T: +31 621388567

ORCID: 0000000334356964

Author addresses

[a]Delft University of Technology, Department of Water Management, Stevinweg 1, 2628 CN Delft, the Netherlands

[b]Veolia Water Technologies Techno Center Netherlands B.V. - Biothane, Tanthofdreef 21, 2623 EW Delft, The Netherlands

Supplementary material

Table S_1 VFA concentration data (part 1)

		CAS														
Stage	Time (day)	C2	C3	i-C4	C4	i-C5	C5	C6	Tot VFA in COD - CAS	C2	C3	i-C4	C4	i-C5	C5	C6
		60.052	74.08	88.11	88.11	102.13	102.13	116.158	mg/L	1.07	1.51	1.82	1.82	2.04	2.04	2.2
		mg/L	mg/L	mg/L	mg/L	mg/L	mg/L	mg/L		%	%	%	%	%	%	%
I	7	1025	315	185	260	315	165	0	3362	33%	14%	10%	14%	19%	10%	0%
	8	440	175	96	140	130	67	0	1566	30%	17%	11%	16%	17%	9%	0%
	9	788	623	195	254	433	196	0	3884	22%	24%	9%	12%	23%	10%	0%
	14	525	517	129	130	217	209	0	2683	21%	29%	9%	9%	17%	16%	0%
	16	503	442	98	88	142	218	0	2279	24%	29%	8%	7%	13%	20%	0%
	20	695	395	172	289	340	93	0	3062	24%	19%	10%	17%	23%	6%	0%
	23	651	128	105	228	129	31	0	1822	38%	11%	10%	23%	14%	3%	0%
	28	552	165	88	113	116	133	0	1714	34%	15%	9%	12%	14%	16%	0%
	30	895	228	175	207	398	336	0	3495	27%	10%	9%	11%	23%	20%	0%
	34	1163	657	265	299	602	61	0	4615	27%	21%	10%	12%	27%	3%	0%
	36	1108	515	237	301	529	472	0	4984	24%	16%	9%	11%	22%	19%	0%
	42	661	223	117	183	269	17	0	2173	33%	15%	10%	15%	25%	2%	0%
	44	1086	369	204	233	458	44	0	3539	33%	16%	10%	12%	26%	3%	0%
	49	1167	269	199	291	417	47	0	3493	36%	12%	10%	15%	24%	3%	0%
	51	1046	215	188	257	426	44	0	3213	35%	10%	11%	15%	27%	3%	0%
	56	1026	150	176	273	392	330	0	3614	30%	6%	9%	14%	22%	19%	0%
	58	1108	256	202	282	432	367	0	4083	29%	9%	9%	13%	22%	18%	0%
	64	1361	191	245	336	586	298	0	4605	32%	6%	10%	13%	26%	13%	0%
	70	1686	119	233	485	537	442	13	5316	34%	3%	8%	17%	21%	17%	1%
	72	1506	123	231	373	513	452	13	4894	33%	4%	9%	14%	21%	19%	1%
	84	1435	161	219	419	591	512	0	5190	30%	5%	8%	15%	23%	20%	0%
II	90	1316	94	34	183	67	71	0	2227	63%	6%	3%	15%	6%	6%	0%
	91	1243	138	27	241	55	73	7	2300	58%	9%	2%	19%	5%	6%	1%
	93	1193	461	27	302	52	98	8	2895	44%	24%	2%	19%	4%	7%	1%
	97	1808	216	91	617	194	203	17	4399	44%	7%	4%	26%	9%	9%	1%
	100	1294	228	72	638	151	162	14	3686	38%	9%	4%	31%	8%	9%	1%
	105	1155	471	62	443	136	164	12	3506	35%	20%	3%	23%	8%	10%	1%
	107	1071	203	47	583	98	126	12	3082	37%	10%	3%	34%	7%	8%	1%
	112	1339	322	77	571	168	210	23	3918	37%	12%	4%	27%	9%	11%	1%
	114	1149	227	61	658	132	193	22	3592	34%	10%	3%	33%	7%	11%	1%
	119	953	452	100	453	216	357	126	4154	25%	16%	4%	20%	11%	18%	7%
	121	1067	254	79	576	169	269	82	3791	30%	10%	4%	28%	9%	14%	5%
	125	1900	146	101	448	218	223	31	4222	48%	5%	4%	19%	11%	11%	2%
	126	2135	139	91	386	196	194	21	4203	54%	5%	4%	17%	9%	9%	1%
	128	1833	314	83	456	179	183	13	4184	47%	11%	4%	20%	9%	9%	1%
	132	1994	244	95	355	203	197	16	4171	51%	9%	4%	15%	10%	10%	1%

Table S_1 VFA concentration data (part 2)

	LAC														
Stage	C2	C3	i-C4	C4	i-C5	C5	C6	Tot VFA in COD - LAC	C2	C3	i-C4	C4	i-C5	C5	C6
	60.1	74.1	88.1	88.1	102.1	102.1	116.2	mg/L	1.07	1.51	1.82	1.82	2.04	2.04	2.2
	mg/L	mg/L	mg/L	mg/L	mg/L	mg/L	mg/L		%	%	%	%	%	%	%
I	365	125	6	535	0	18	100	1821	21%	10%	1%	53%	0%	2%	12%
	420	23	0	325	0	18	95	1321	34%	3%	0%	45%	0%	3%	16%
	580	130	0	490	0	27	126	2041	30%	10%	0%	44%	0%	3%	14%
	511	104	0	253	0	9	18	1222	45%	13%	0%	38%	0%	2%	3%
	556	93	0	216	0	0	0	1128	53%	12%	0%	35%	0%	0%	0%
	600	34	0	75	0	0	0	830	77%	6%	0%	16%	0%	0%	0%
	710	53	5	126	0	0	0	1078	70%	7%	1%	21%	0%	0%	0%
	513	78	0	111	0	0	0	869	63%	14%	0%	23%	0%	0%	0%
	665	145	0	135	0	0	0	1176	60%	19%	0%	21%	0%	0%	0%
	600	97	0	400	0	0	0	1516	42%	10%	0%	48%	0%	0%	0%
	710	86	0	225	0	0	0	1299	58%	10%	0%	32%	0%	0%	0%
	695	145	0	290	0	0	0	1490	50%	15%	0%	35%	0%	0%	0%
	670	40	0	275	0	0	0	1278	56%	5%	0%	39%	0%	0%	0%
	795	155	0	255	0	0	0	1549	55%	15%	0%	30%	0%	0%	0%
	590	99	0	230	0	0	0	1199	53%	12%	0%	35%	0%	0%	0%
	585	97	0	420	0	0	0	1537	41%	10%	0%	50%	0%	0%	0%
	1035	165	0	515	0	0	0	2294	48%	11%	0%	41%	0%	0%	0%
	680	195	0	355	0	0	0	1668	44%	18%	0%	39%	0%	0%	0%
	700	155	0	385	0	6	0	1696	44%	14%	0%	41%	0%	1%	0%
	800	170	0	520	0	0	0	2059	42%	12%	0%	46%	0%	0%	0%
	540	99	0	375	0	0	0	1410	41%	11%	0%	48%	0%	0%	0%
II	1055	134	16	406	32	16	0	2197	51%	9%	1%	34%	3%	2%	0%
	1021	116	14	544	29	18	10	2403	45%	7%	1%	41%	2%	2%	1%
	990	92	16	588	33	20	53	2522	42%	6%	1%	42%	3%	2%	5%
	1249	124	61	656	126	50	131	3482	38%	5%	3%	34%	7%	3%	8%
	1195	117	52	530	107	109	141	3267	39%	5%	3%	30%	7%	7%	9%
	958	321	84	669	185	204	126	3951	26%	12%	4%	31%	10%	11%	7%
	945	285	72	733	158	191	115	3870	26%	11%	3%	34%	8%	10%	7%
	1213	165	100	807	216	187	155	4362	30%	6%	4%	34%	10%	9%	8%
	1266	181	99	794	215	188	154	4414	31%	6%	4%	33%	10%	9%	8%
	1002	477	105	623	222	237	114	4302	25%	17%	4%	26%	11%	11%	6%
	1004	430	100	729	215	237	94	4361	25%	15%	4%	30%	10%	11%	5%
	1384	181	82	604	176	167	119	3964	37%	7%	4%	28%	9%	9%	7%
	1441	149	66	641	140	135	98	3831	40%	6%	3%	30%	7%	7%	6%
	1354	110	52	630	108	109	85	3488	42%	5%	3%	33%	6%	6%	5%
	1620	126	73	426	154	145	61	3575	48%	5%	4%	22%	9%	8%	4%
